# 

**Published:** 1898-02-19

**Authors:** 


					]
I
THE HOSPITAL. ? The standard of highest purity."?Lancet. Feb. 19th, 1898.
CADBURY'S M to* CADBURY'S
COCOA nr jMr?tMI Mm COCOA
ABSOLUTELY PURE. Js* ? ? DRUGS OR ALKALIES USED,
0
^?- 595; Vol. XXm. r^g^pen] SATTODAT,
Feb. 19th, 1898.
[Snhsoripticin^Terms,] pRICE TWOPENCE.

				

